# Cancer Collusion?: Dietary Fat May Modify Dioxin-Induced Mammary Cancer Risk

**DOI:** 10.1289/ehp.118-a217b

**Published:** 2010-05

**Authors:** M. Nathaniel Mead

**Affiliations:** **M. Nathaniel Mead**, a science writer living in Durham, NC, has written for *EHP* since 2002

Some human and animal studies have linked early-life exposure to the endocrine-disrupting chemical 2,3,7,8-tetrachlorodibenzo-*p*-dioxin (TCDD) with an increased susceptibility to breast cancer. Dietary fat has been posited as another potential risk factor for breast cancer, possibly acting through the estrogen pathway. A new animal study suggests a high-fat diet may alter estrogen metabolism, thereby modifying the effects of maternal exposure to TCDD and increasing mammary cancer risk in the next generation **[*EHP*** **118:596–601; La Merrill et al.]**.

One group of pregnant female FVB/NJ mice (a TCDD-responsive mouse strain) received an olive oil/toluene blend with TCDD; another received an equivalent volume of olive oil/toluene without TCDD. Their female offspring were randomly assigned to either a low-fat or high-fat diet and exposed to the carcinogen 7,12-dimethyl-benz[*a*]anthracene (DMBA) at days 35, 49, and 63 after birth in order to initiate mammary tumors. A second cohort of female offspring was treated identically until either day 35 or 49, when morphologic and molecular analyses of their mammary glands were performed.

Maternal TCDD exposure was associated with a doubling of mammary tumor incidence only in offspring fed the high-fat diet. In contrast, no mammary tumors arose in mice exposed to TCDD *in utero* and fed the low-fat diet. Whereas one-third of TCDD-unexposed litters fed a high-fat diet had DMBA-induced mammary lesions, every litter exposed to both TCDD and a high-fat diet developed mammary lesions.

Previous animal studies had shown that prenatal exposure to TCDD alters mammary gland differentiation and increases susceptibility to mammary cancer. However, this is the first to show that TCDD may interact with a high-fat diet during pregnancy and early life. The researchers propose that a high-fat diet may boost sensitivity to maternal TCDD exposure by altering estrogen metabolism.

The new findings highlight a possible mechanism that may explain epidemiologic data separately linking early-life TCDD exposure and high-fat diets to increased breast cancer risk in humans. In the present study, TCDD exposure *in utero* combined with a high-fat diet was also associated with increased expression of *Cyp1b1* and decreased expression of *Comt* in mammary tissue. Human studies have suggested that diminished *COMT* expression and increased *CYP1B1* expression are associated with increased risk of breast cancer and other estrogen-responsive cancers, perhaps by increasing levels of estrogen metabolites that can contribute to carcinogenesis by damaging DNA. They also note that obesity may affect breast cancer risk because TCDD persists in adipose tissue, including that in the breast.

